# Kinetics of Phase Transitions in Amorphous Carbamazepine: From Sub-*T_g_* Structural Relaxation to High-Temperature Decomposition

**DOI:** 10.3390/ijms26136136

**Published:** 2025-06-26

**Authors:** Roman Svoboda, Adéla Pospíšilová

**Affiliations:** Department of Physical Chemistry, Faculty of Chemical Technology, University of Pardubice, Studentská 573, 532 10 Pardubice, Czech Republic

**Keywords:** carbamazepine, structural relaxation, crystal growth, thermal decomposition

## Abstract

Thermokinetic characterization of amorphous carbamazepine was performed utilizing non-isothermal differential scanning calorimetry (DSC) and thermogravimetry (TGA). Structural relaxation of the amorphous matrix was described in terms of the Tool–Narayanaswamy–Moynihan model with the following parameters: Δh* ≈ 200–300 kJ·mol^−1^, *β* = 0.57, *x* = 0.44. The crystallization of the amorphous phase was modeled using complex Šesták–Berggren kinetics, which incorporates temperature-dependent activation energy and degree of autocatalysis. The activation energy of the crystal growth was determined to be >320 kJ·mol^−1^ at the glass transition temperature (*T_g_*). Owing to such a high value, the amorphous carbamazepine is stable at *T_g_*, allowing for extensive processing of the amorphous phase (e.g., self-healing of the quench-induced mechanical defects or internal stress). A discussion was conducted regarding the converse relation between the activation energies of relaxation and crystal growth, which is possibly responsible for the absence of sub-*T_g_* crystal growth modes. The high-temperature thermal decomposition of carbamazepine proceeds via multistep kinetics, identically in both an inert and an oxidizing atmosphere. A complex reaction mechanism, consisting of a series of consecutive and competing reactions, was proposed to explain the second decomposition step, which exhibited a temporary mass increase. Whereas a negligible degree of carbamazepine degradation was predicted for the temperature characteristic of the pharmaceutical hot-melt extrusion (~150 °C), the degradation risk during the pharmaceutical 3D printing was calculated to be considerably higher (1–2% mass loss at temperatures 190–200 °C).

## 1. Introduction

Carbamazepine (CBZ, 5H-dibenz[b,f]azepine-5-carboxamide, C_15_H_12_N_2_O, m.w. = 236.27 g·mol^−1^) is an active pharmaceutical ingredient (API) primarily used to control certain types of seizures, i.e., the focal or tonic–clonic seizures, through the stabilization of the hyperexcited nerve membranes, inhibition of the repetitive neuronal discharges, and reduction in the synaptic transmission [1,2,3,4]. The same mechanism is also utilized for the treatment of trigeminal neuralgia and the management of acute manic and mixed episodes (it acts as a mood stabilizer for bipolar I disorder) [5,6,7]. Due to its poor solubility in water (~17 mg·L^−1^ [8,9]), the amorphous form of CBZ is considered in modern formulations as a means to increase its bioavailability through a faster dissolution rate and overall improved aqueous solubility. Note that CBZ is a Biopharmaceutics Classification System (BCS) Class II drug, i.e., API, with low solubility and high permeability [10]. The disordered amorphous state, with its lower lattice energy and higher free energy, also has drawbacks, namely, low thermal stability, often leading to fast and uncontrolled recrystallization (full or partial) of the amorphous material [11,12,13]. CBZ is listed in the Taylor classification [14] of the crystallization tendency under class 2, i.e., as a compound that does not crystallize during cooling from its melt at 20 °C·min^−1^ but crystallizes during the subsequent reheating of the formed amorphous phase at 10 °C·min^−1^. For CBZ, the required cooling rate is indeed quite high (borderline 20 °C·min^−1^), which means that the melt-quench-based preparation of the amorphous CBZ in large batches is rather difficult. In such cases, the center of the liquid mass is often cooled at a slower rate due to insufficient heat transfer, and since CBZ can crystallize not only from the surface defects but also from homogeneously dispersed volume-located nuclei, the recrystallization is initiated from the center of the amorphous bulk [15,16]. This is probably why research on the crystal growth kinetics and overall thermal stability of (amorphous) CBZ is very scarce.

Based on a thorough literature survey, only a few research papers were found that reported the thermo-kinetic data of (amorphous) CBZ. Structural relaxation in amorphous CBZ was studied utilizing temperature-modulated differential scanning calorimetry (DSC) and dielectric spectroscopy in [17]; the kinetic fragility [18] of CBZ was determined to be 96 ± 1, and the Kohlrausch distribution parameter [19,20] *β* = 0.42. The shift in quench-cooled CBZ glass transition temperature (*T_g_*) with the applied heating rate was measured in [16] to determine the kinetic fragility of 62.5 and the activation energy of structural relaxation, Δh* = 396 kJ·mol^−1^. The kinetics of CBZ crystallization from the amorphous state were studied in [21], where activation energies of 107 and 185 kJ·mol^−1^ were reported for the high- and low-temperature regions, respectively. The attempt to describe the data using Avrami kinetics [22,23,24] led to the conclusion that the crystal growth is closest to three-dimensional crystal formation, proceeding from continually forming nuclei. The microscopically determined crystal growth rates were reported in [25] for the 60–190 °C temperature range, with growth rates ranging between 10^−10^ and 10^−4^ m·s^−1^. The thermal decomposition kinetics of CBZ was studied in [26], reporting the corresponding activation energy of 93 ± 2 kJ·mol^−1^.

As mentioned above, the current state of knowledge regarding the thermal behavior of amorphous CBZ is largely unsatisfactory, particularly with respect to its potential for mass production in an amorphous form and its subsequent processing and storage. The low-temperature thermal stability of amorphous CBZ needs to be considered from several points of view: (1) the standard crystallization processes (occurring above *T_g_*) dictate the limits for various processing conditions, including the annealing at *T_g_*, leading to the self-healing of surface defects and micro-cracks that may accelerate the recrystallization [27,28]; (2) susceptibility to the enhanced surface growth or rapid diffusion-less growth modes [29,30,31], which occur primarily below *T_g_* as a consequence of the increased molecular mobility at surface and along the defects; (3) intensity and temperature dependence of long-term structural relaxation processes [32,33], which can be the source of internal stresses, again leading to the increased nucleation and/or growth rates [34]. Similarly to the low-temperature stability, also the high-temperature stability of the CBZ melt is crucial in modern medicinal applications, where the APIs (either in crystalline or in the stabilized amorphous form) are dispersed in the suitable pharmaceutical polymers, extruded and 3D-printed into personalized tablets, offering the possibility to tailor the parameters of the controlled release mechanism [35,36,37]. In these applications, the API must be sufficiently thermally stable to withstand the increased temperatures required to soften or melt the (dominant) polymeric portion of the formulation matrix [38,39].

In the present paper, the questions related to the processing conditions of (amorphous) CBZ will be investigated using combined thermo-analytical, microscopic, and spectroscopic techniques. The detailed kinetics of all relevant processes (crystal growth, structural relaxation, and thermal decomposition) will be determined and discussed. In addition to the practical outcomes (kinetic predictions and consequent recommendations regarding CBZ processing), the academic scrutiny will focus on the mutual relationships between relaxation and crystal growth phenomena, as recently reported in detail for other archetypal APIs [40,41,42,43]. In this regard, the amorphous CBZ represents an undocumented case of a material with very low thermal stability, despite its high *T_g_*.

## 2. Results

The thermal behavior of amorphous CBZ was investigated using differential scanning calorimetry (DSC), thermogravimetry (TGA), optical microscopy, and Raman spectroscopy. The present section will report on these results with the following structure: (1) data from the DSC crystallization experiments, (2) data from the DSC structural relaxation experiments, (3) TGA data on thermal decomposition, and (4) microscopic and spectroscopic characterization.

Despite CBZ being classified as a “class 2” drug according to [14] (meaning that it should not crystallize during cooling of melt at cooling rate *q*^−^ = 20 °C·min^−1^), it exhibited a relatively high tendency toward crystallization when being attempted to prepare amorphous ingot from larger batch via the classical batch melt-quench method. In addition, thermal instability of CBZ was also observed during its preparation, where slow and/or prolonged heating above *T_m_* led to a change in the liquid color (from clear to yellow). Hence, a special standardized procedure had to be adopted, with each amorphous CBZ sample being prepared separately and directly in the DSC pan. The crystalline CBZ powder was accurately weighed into the DSC pan, which was then heated to 200 °C for 20 s and immediately quenched via a heat sink. In this way, fully amorphous samples were always prepared (as confirmed by optical microscopy) with no change in color.

**Figure 1:**
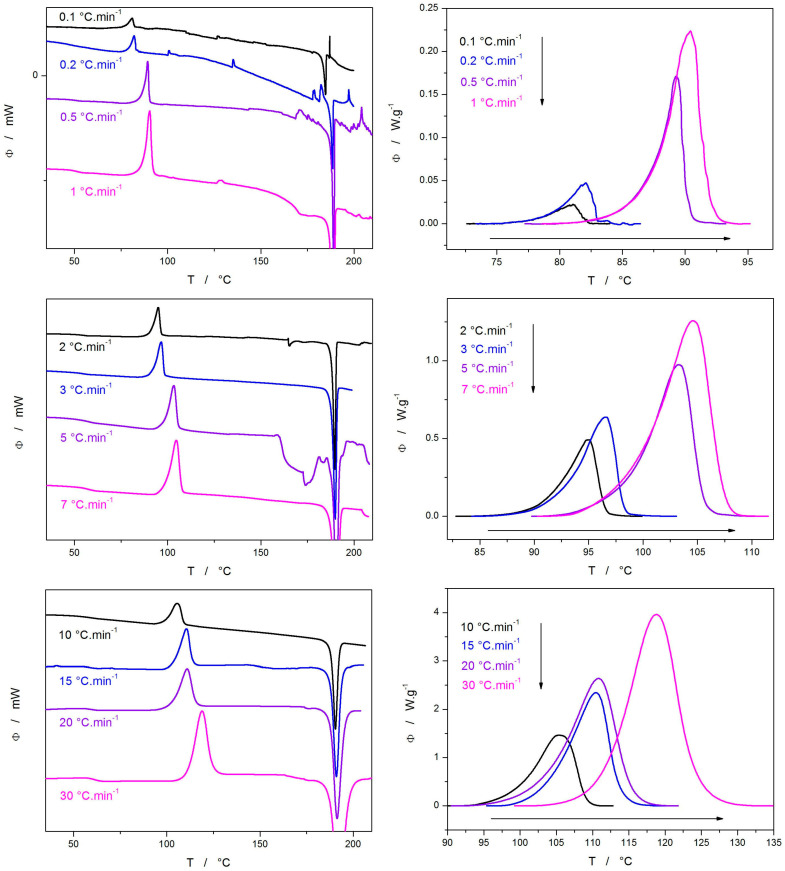
Raw DSC heating curves (**right column**); crystallization signals with subtracted thermokinetic background (**left column**). Exothermic effects evolve in an upward direction.

The overall thermal characterization of amorphous CBZ samples, prepared individually by melt-quenching within the DSC pan, is depicted in Figure 1. The left column depicts the raw DSC data obtained at different heating rates *q*^+^. The first identifiable thermokinetic effect is the glass transition, positioned at ~55–65 °C. Since the samples were measured immediately after the melt-quench, no significant relaxation had time to occur, resulting in the practical absence of the relaxation peak (which typically appears just above the glass transition midpoint in the undercooled liquid region). It is very important to mention that no recognizable exothermic signal was observed in the vicinity of *T_g_*, which would indicate a manifestation of the rapid diffusionless glass–crystal (GC) growth mode. It was shown recently that the GC growth can be directly monitored by DSC in the case of certain low-molecular organic glasses (e.g., nifedipine [40] or griseofulvin [41]). However, the GC growth signal was always obtained only in the case of finely ground or milled powders with large amounts of mechanically induced defects (edges, microcracks, etc.), which serve as nucleation centers and propagate the GC growth. The present samples were in the form of bulk “pieces” with several cracks (formed due to the internal stress induced by thermal contraction during rapid cooling). Such a low level of surface (as well as internal) damage evidently does not suffice to give basis to a DSC-detectable amount of the GC growth.

Standard crystallization occurs in CBZ between 75 and 125 °C (depending on *q*^+^) and manifests as a simple exothermic peak with *q*^+^-dependent asymmetry. At lower *q*^+^, the DSC crystallization peaks are strongly negatively asymmetric, exhibiting skewing to higher *T*/*α* (temperature/degree of conversion). At higher *q*^+^, the peaks become more symmetric. Importantly, even at extremely low *q*^+^, the CBZ crystallization peaks are uniform, unlike those obtained for larger grains of, e.g., indomethacin [42], griseofulvin [41], nifedipine [40], or nimesulide [43]. This indicates that (contrary to those other amorphous APIs) crystal growth in CBZ has to originate from a large number of volume-located nuclei, rather than solely from the surface defects that are scarce for bulk samples, which then, in turn, leads to independent formation of several separated crystallization signals. On the other hand, the endothermic melting peak of CBZ (onset at ~188 °C) gets rather complex at low *q*^+^, exhibiting not only several adjacent endothermic peaks combined with exothermic recrystallization signals but also a number of additional parasitic signals and increased data scatter. Whereas the former indicates polymorphic transformations occurring in the sample (indicating the formation of different minor phases at low *q*^+^), the latter points to thermal degradation/decomposition taking place at temperatures close to the melting point, *T_m_*, as described above.

Note that CBZ is known to have numerous polymorphic phases [44,45], with the most common ones being form I (*T_m_* ≈ 192–193 °C), form II (*T_m_* ≈ 179–180 °C), form III (*T_m_* ≈ 174–176 °C), and form IV (*T_m_* ≈ 181–182 °C). The present amorphous samples were prepared from crystalline powder, a mixture of forms I and III. At higher *q*^+^, the amorphous CBZ unambiguously preferably crystallizes into form I (as the *T_m_ons_* is almost invariable at ~188 °C), with only traces of form III being present—see the very small melting pre-peak at ~173 °C. With decreasing *q*^+^, the melting signals become more complex, showing occasional stronger form III pre-peaks. It has to be, however, borne in mind that at low *q*^+^ (≤1 °C·min^−1^), the time spent near *T_m_* is high enough for CBZ to start to decompose. The partial degradation of CBZ is certainly responsible for the numerous scatter near *T_m_* that occurs for the measurements performed at *q*^+^ ≤ 0.5 °C·min^−1^. Polymorphs-wise, it is probable that instead of the crystal growth suddenly changing at 0.1 °C·min^−1^ from form I to form IV (as suggested by the position of the melting peak at 183 °C), the CBZ decomposition proceeded below *T_m_* to such a large extent that it influenced the melting peak as a whole through the well-known impurity-induced melting point depression. Base thermal characteristics evaluated from the DSC curves are listed in Table 1.

In order to extract the pure crystallization signals from the DSC data, the tangential area-proportional baseline [46] (practically the only physically meaningful subtraction of the thermokinetic background) was applied:

(1) $$B\left(T\right)=\left(1-\alpha \left(T\right)\right)\times \left(z_{0,r}+z_{1,r}\times T\right)+\alpha \left(T\right)\times \left(z_{0,p}+z_{1,p}\times \left(T_{f}-T\right)\right)$$

where *B*(*T*) is the temperature dependence of the baseline curve, *α* is the degree of conversion, *z_*0*,r_,* and *z_*1*,r_* are the coefficients characterizing the tangent going through the starting point (in the reactants area), *z_*0*,p_* and *z*_1,*p*_ are the coefficients characterizing the tangent going through the endpoint (in the products area), and *T_f_* is the endpoint temperature. Since each sample was prepared separately and the preparations combined with immediate measurements were conducted in random order (not correlated with increase or decrease in *q*^+^ applied during the DSC heating scan), the almost exactly regular pairing of the onset edges of the depicted DSC curves seems to be either an odd coincidence or a material characteristic associated with nucleation occurring during heating of the amorphous phase. This phenomenon will be further discussed in Section 3.2, where the kinetic analysis of the pure crystallization signals will be performed.

**Figure 2:**
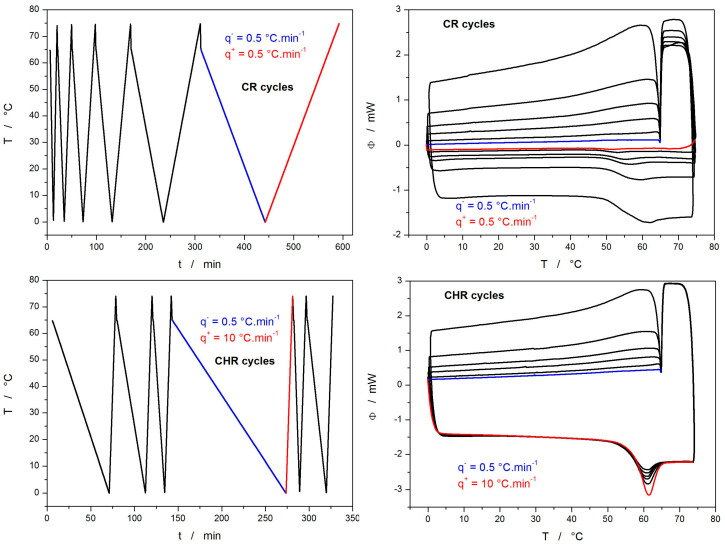
Temperature programs for the CR and CHR structural relaxation experiments (**left column**), and the corresponding DSC responses (**right column**). Exothermic effects evolve in an upward direction.

Following the overall characterization of the CBZ amorphous samples, this research was further focused on the processes that were not fully described by the simple heating scans depicted in Figure 1. The first such process, essential for the behavior of the amorphous phase, the position of *T_g_*, and the consequent growth of crystals, is structural relaxation, i.e., the reorganization of the amorphous phase below *T_g_* at long timescales [32,33]. The relaxation measurements were performed in accordance with the standard procedure [47], utilizing the constant ratio (CR) and constant heating rate (CHR) cyclic experiments. During these measurements, the same sample (one for each set of cycles) is repeatedly cycled through the glass transition region at different rates. In CR cycles, the heating rate is always the same as was in the previous cooling step; during the CHR cycles, *q*^+^ is constant (10 °C·min^−1^), and only *q*^−^ varies. As the sample needs to be equilibrated (usually 30–60 s) above *T_g_* to achieve a reproducible state of the undercooled liquid, and the measurement needs to be performed to a high enough temperature for the temperature dependence of heat capacity (*c_p_*-*T*) to be extrapolatable, the low thermal stability of the CBZ amorphous phase started to play a role. The first series of measurements was performed in the temperature range of 0–75 °C. However, the repeated equilibration of the sample at 75 °C (despite being for only 30 s) led to a quick degradation of the amorphous phase—an almost complete crystallization occurred within a few cycles. Hence, the temperature program of a cycle was adjusted as follows: (1) cooling from 65 to 0 °C, (2) heating from 0 to 75 °C, (3) immediate cooling from 75 to 65 °C, and (4) equilibration for 60 s. The CR and CHR cycles temperature programs and the corresponding DSC data are shown in Figure 2; the applied cooling rates were *q*^−^ = 0.5, 1, 2, 3, 5, and 10 °C·min^−1^ (the cooling from 75 to 65 °C was always 10 °C·min^−1^).

The CR cycles were performed in the order of decreasing *q*^−^ (as shown in Figure 2). During the last cycle, where the last step of the whole CR series was heating of the sample to 75 °C at 0.5 °C·min^−1^, the sample already started to crystallize—as is indicated by the exothermic signal on the red curve (with the onset at ~70 °C) of the corresponding graph. Since it was the last cycle, and the crystallization occurred above *T_g_*, the related evaluation of the structural relaxation kinetics was not affected. It is, however, noteworthy that for the standard heating scan of a fresh amorphous sample, the onset of the crystallization is at ~80 °C (see Figure 1). The accelerated crystal growth observed during the last heating scan of the CR cycles indicates a significant amount of nuclei being formed during the CR cycles. With this knowledge, the CHR cycles series was performed randomly with respect to the order of the applied *q*^−^—as shown in Figure 2. This was conducted primarily to identify any potential trends in the *c_p_*-*T* dependences that would indicate the influence of nucleation on the relaxation response. No such feature was observed, and the *c_p_*-*T* dependence was perfectly reproducible in both glassy and undercooled liquid regions. It is, however, very important to mention that the overall relaxation response (namely the width of the relaxation peak) was somewhat different for the cycle characterized by *q*^+^ = 10 °C·min^−1^ and *q*^−^ = 10 °C·min^−1^—this cycle was the only one present within both cycle sets; the comparison of the two curves is shown in the Supplementary Materials. Two explanations come to mind here: (1) the other cycles preceding the 10 and 10 cycle during the CHR cycles stabilized the glassy structure, resulting in a narrower relaxation peak. Nonetheless, as the erasure of thermal history also preceded the 10 and 10 cycle performed during the CR cycles, the “stabilization” of the glassy matrix should have already been finished in this case. Note here that an instantaneous achieving of thermal and structural pseudo-equilibrium in the undercooled liquid state is an inherent and widely accepted feature of the TNM kinetics (at least for inorganic and low-molecular organic glasses) [18]. (2) Very recently, Prof. Mark Ediger (University of Wisconsin–Madison) reported [48] on a newly discovered phenomenon in low-molecular glasses—a polymorphism in the amorphous undercooled liquid state (above but near *T_g_*). This would indeed explain the small difference in the relaxation peak shape between the two sets (CR and CHR cycles). Note, however, that these findings are still very new and not yet verified by other independent laboratories at the time of the present paper submission.

**Figure 3:**
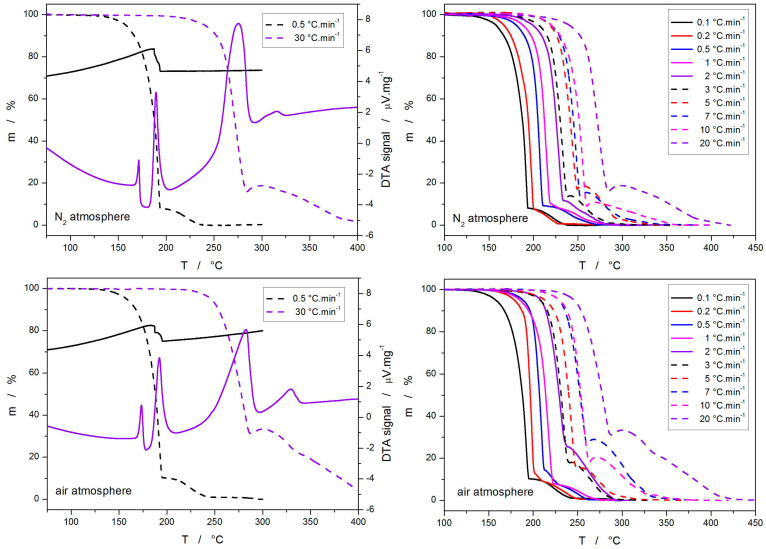
Selected joint DTA-TGA signals obtained for the CBZ decomposition in N_2_ and air atmospheres (**left column**)—exothermic effects evolve in a downward direction; the (**right column**) depicts full sets of TGA measurements.

The second process observed, but not fully recorded by the DSC heating scans shown in Figure 1, is the thermal decomposition of CBZ. For this reason, supplemental joint TGA-DTA measurements were performed in open pans to monitor the mass loss (and the associated heat changes) corresponding to the degradation of the as-purchased CBZ; the data are shown in Figure 3. The main decomposition step is followed by a subsequent (much smaller) step, the magnitude of which increases with *q*^+^. At higher *q*^+^, a small mass increase also appears to be associated with the transition between the two mass-loss steps. As is apparent, the signals are extremely similar in both N_2_ and air atmospheres, indicating either that the sample generates its own oxidative atmosphere or that no oxidative reaction occurs at all. Since all heat signals associated with the decomposition steps are endothermic, the latter is a much more probable option. The main difference between the decomposition processes proceeding in the two atmospheres is the significantly smaller magnitude of the first/main decomposition step—approx. 85% in N_2_ atmosphere vs. approx. 70% in an air atmosphere at higher *q*^+^. On the contrary, a major difference is observed between the heat flow signals recorded at low and high *q*^+^. However, at high *q*^+^, the sample is fully melted, and the loss of the mass follows from the liquid state, at low *q*^+^, the decomposition occurs before the sample is melted, and the melting process can thus be altered or even suppressed (as observed for the case of the form III melting pre-peak). It is noteworthy that the much higher decomposition intensity (altering the CBZ structure and hindering the melting processes) recorded by the joint TGA-DTA is caused by the fact that the as-purchased CBZ was in the form of fine powder with a large surface and, hence, high reactivity. In comparison, the DSC records from Figure 1 were obtained for the melt-quenched bulk samples, which have a very low surface-to-volume ratio, and thus, a much lower degree of decomposition was achieved at the comparative combinations of *T* and *q*^+^. The thermal decomposition of CBZ proceeds as a combination of a split-off into the isocyanic acid and iminostilbene and the sublimation of these products, as well as of the original CBZ [26,49,50]. In addition, small amounts of CO_2_, H_2_O, and NH_3_ can be detected during CBZ decomposition [26,50]. It can be thus assumed that the sublimation will more dominantly occur at lower *q*^+^ [49], whereas the further decomposition into smaller fragments (aromatic hydrocarbons) will be more probable at higher *q*^+^, where higher *T* is achieved before the sample is gasified. The observed transient increase in sample mass can then be explained by the reaction and subsequent condensation of the reactive radical or carbocation fragments/intermediates [26,49,50].

**Figure 4:**
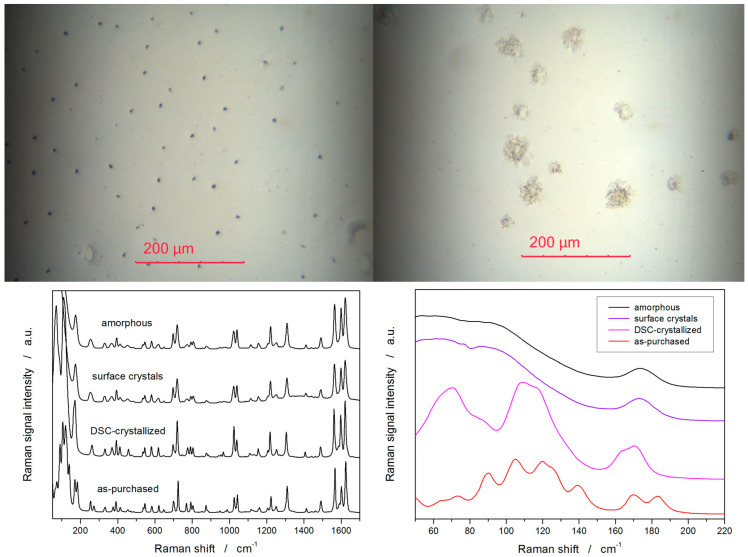
The top left and right micrographs show the amorphous CBZ sample heated to 80 °C for 5 and 10 min, respectively. Bottom graphs show the Raman spectra (full and zoomed-in on the low-phonon part) of the amorphous, as-purchased, DSC crystalized, and surface-crystallized (crystals from the micrograph above) CBZ samples.

In addition to the thermoanalytical techniques, transmission optical microscopy was employed to monitor the character of the present nucleation and crystal growth processes. The two micrographs depicted in Figure 4 show the melt-quenched sample (identical to those used for the DSC measurements) after 5 and 10 min at 80 °C. The first micrograph depicts the nucleation density, which originates mainly from the melt quench procedure—the majority of these small crystals are located within the bulk volume of the sample, rather than on the surface or at the substrate/sample interface. The second micrograph shows well-grown crystals, the diameter of which ranges from ~25 to 55 μm. The large variation in the crystal diameters indicates that continuous nucleation proceeds at 80 °C simultaneously with the growth of pre-existing nuclei formed during the melt quench. It is also clear that the largest crystals in the second micrograph are certainly much more than twice as large compared to the crystals from the first micrograph—this indicates that the crystal growth started at 80 °C only after a certain period of time, just before the 5 min mark, giving the rough estimate of the crystal growth rate at this temperature to ~10 μm·min^−1^.

Additional characterization was also performed using Raman microscopy; four archetypal spectra are shown in Figure 4. Since the Raman spectra for the amorphous and crystalline CBZ are very similar, main attention needs to be paid to the low-energy phonon region and the Rayleigh edge. As the first crystals formed at the sample surface are very thin (with the underlying amorphous phase), the corresponding confocal Raman signal is practically indistinguishable from the pure amorphous sample. Considering the spectral profiles of different CBZ polymorphic forms [51,52], the fully DSC-crystallized CBZ samples then show clear evidence of the dominant presence of the polymorphic form I, with only very weak traces of the form III polymorph (as also evidenced by the DSC data in Figure 1). On the other hand, the Raman spectrum of the as-purchased CBZ confirms that a dominant portion of the material is in the form of III. This is crucial information for the correct interpretation of the DTA data depicted in Figure 3: despite the melting pre-peak (corresponding to form III) being much smaller in magnitude compared to the dominant melting signal of form I, it is followed by the exothermic signal indicating the recrystallization of form I from the melt (corresponding to the monotropic polymorphism). The overlap of the endothermic pre-peak melting and the consequent exothermic recrystallization masks the true magnitude of both effects. As evidenced by the Raman spectroscopy, the majority of the as-purchased CBZ was indeed a form III polymorph (a similar conclusion was also reached based on the XRD analysis reported in [16]). This finding is important from the synthesis (melt-quench) point of view, confirming that the CBZ powder completely melted during the 20 s at 200 °C before the melt-quench and that no form III nuclei or unmelted crystals remained in the liquefied sample.

## 3. Discussion

The present section will be divided into four subsections, where the first three will report on the kinetics of structural relaxation, crystallization, and thermal decomposition kinetics, and the fourth section will discuss the mutual interrelationships between these phenomena.

### 3.1. Structural Relaxation Kinetics

In the present literature, the calorimetric structural relaxation data are most often described in terms of the phenomenological Tool–Narayanaswamy–Moynihan (TNM) model [53,54,55]:

(2) $$C_{p}^{red}\left(T\right)=\frac{dT_{f}}{dT}=\frac{C_{p}\left(T\right)-C_{pg}\left(T\right)}{C_{pl}\left(T\right)-C_{pg}\left(T\right)}$$

(3) $$\Phi \left(t\right)=\frac{T_{f}\left(t\right)-T_{f}(\infty )}{T_{f}\left(0\right)-T_{f}(\infty )}$$

(4) $$\Phi \left(t\right)=exp\left[-{\left(\int_{0}^{t}\frac{dt}{\tau (T,T_{f})}\right)}^{\beta }\right]$$

(5) $$\tau \left(T,T_{f}\right)=A_{TNM}\times exp\left[x\frac{∆h^{*}}{RT}+(1-x)\frac{∆h^{*}}{RT_{f}}\right]$$

where *C_p_^red^* is the reduced heat capacity function used to normalize the derivative DSC data. Note that *c_pg_*(*T*) and *c_pl_*(*T*) are the temperature dependences of the heat capacity in the glassy and undercooled liquid regions, respectively. *T_f_* then stands for the fictive temperature, i.e., the hypothetical temperature of a liquid with the same density and structural ordering as that of the relaxing glass in the given moment. *Φ*(*t*) is the relaxation function based on the normalized integral relaxation data (in the form of *T_f_* and not d*T_f_*·d*T*^−1^), *t* is time, *τ* is the relaxation time, *β* is the non-exponentiality parameter, *A_TNM_* is the pre-exponential factor, *x* is the non-linearity parameter, ∆h* is the relaxation activation energy, *R* is the universal gas constant, and *T* is temperature. The goal of the present measurements is to determine the four phenomenological parameters, i.e., Δh*, *x*, *β*, and *A_TNM_*.

**Figure 5:**
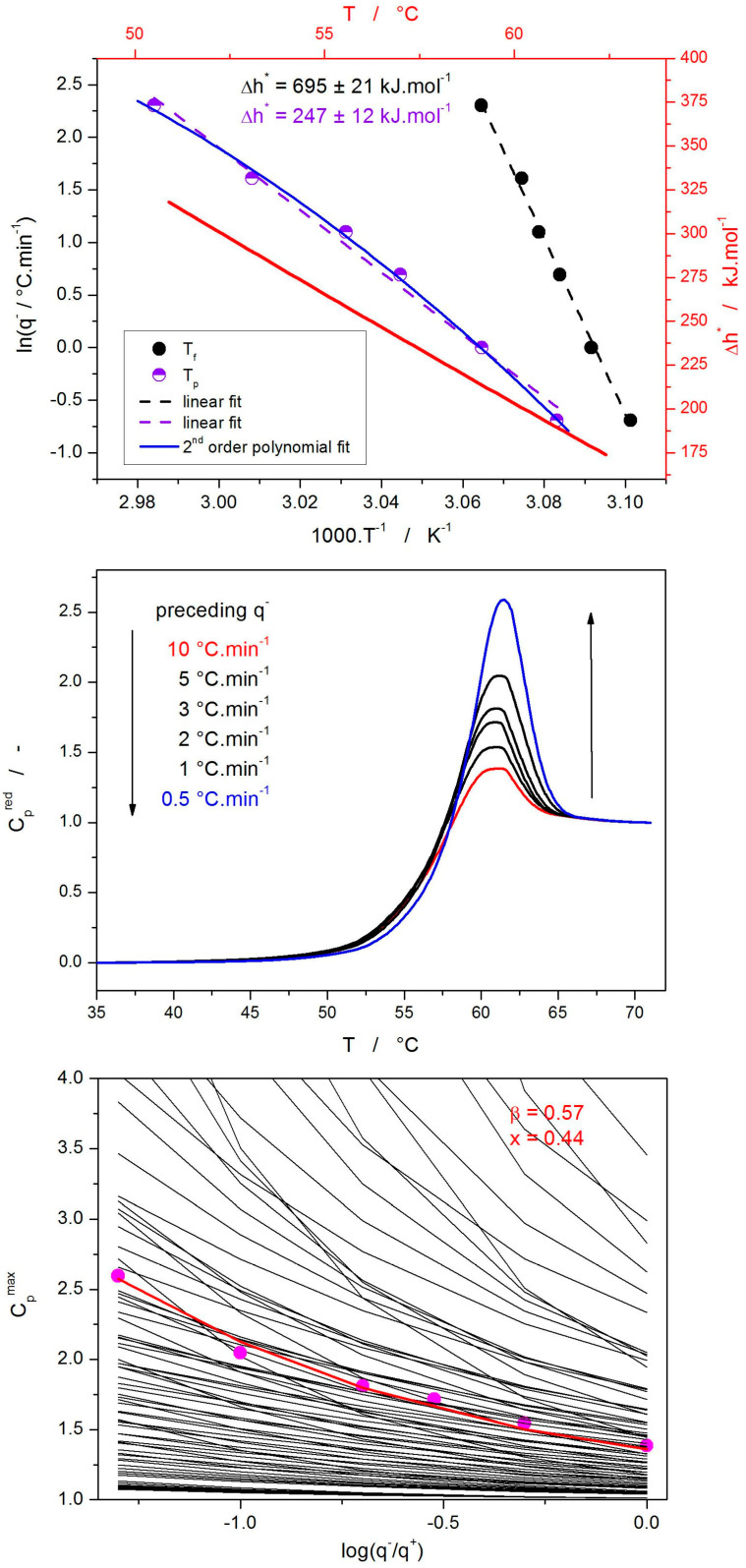
(**Top graph**): Evaluation of Δh* from CR and CHR cycles with the listed values corresponding to the linear fits (bottom and left axes); Δh*-*T* dependence determined from the CR cycles evaluation fit by the 2nd-order polynomial (top and right axes). (**Middle graph**): Normalized set of CHR cycles. (**Bottom graph**): Application of the simulation-comparative method to the present data—points show the experimental data from the middle graph, thin lines depict selected simulated dependences for various β&x combinations, thick red line shows the best correspondence between the experimental and simulated data (*β* = 0.57, *x* = 0.44).

In the first step of the kinetic analysis, the activation energy of structural relaxation needs to be determined. Depending on what temperature program is used (CR or CHR cycles), either the Svoboda [56] (Equations (6) and (7)) or Moynihan [55] (Equation (8)) methodology is used:

(6) $$-\frac{∆h^{*}}{R}={\left[\frac{dln\left|q^{-}\right|}{d(1/T_{p})}\right]}_{q^{-}/q^{+}=const.}$$

(7) $$\begin{array}{c} \left[\left({∆h}_{exp}^{*}/R\right)-\left({∆h}_{true}^{*}/R\right)\right]\times 100\%\\ \;\;\;\;\;\;\;\;\;\;\;\;\;\;\;\;\;\;\;\;\;\;\;\;\;\;\;\;=4.218\times 10^{-5}{\left({∆h}_{true}^{*}/R\right)}^{2}+4.841\times 10^{-2}\left({∆h}_{true}^{*}/R\right)\\ \;\;\;+\left[9.885\times 10^{1}/\left(\left({∆h}_{true}^{*}/R\right)-1.276\right)\right] \end{array}$$

(8) $$-\frac{∆h^{*}}{R}=\frac{dln\left|q^{-}\right|}{d(1/T_{f})}$$

where *q*^−^ is the cooling rate applied just before the heating step, from which either the temperature of the maximum of the relaxation peak *T_p_* or the fictive temperature *T_f_* is evaluated. Note that *T_f_* is usually calculated using the Moynihan equal-area method [56]. The indices “exp” and “true” refer to the experimentally obtained (via Equation (6)) and true values of activation energy Δh*. These evaluations are based on the current data on the structural relaxation of CBZ, as depicted in the top graph of Figure 5. The listed Δh* values were calculated from the linear fits of the two dependencies shown. It is immediately clear that the slopes of both dependencies are vastly different. This is a common issue when the Moynihan method often largely overestimates the true Δh* values [47]. However, even the evaluation from the CR cycles, as determined by Equations (6) and (7), did not yield a uniform Δh* value due to the curvature of the corresponding dependence. Hence, to obtain the most accurate results, a second-order polynomial fit was used, and the Δh*-*T* dependence was calculated from its derivation (in accordance with Equation (6)). In the explored *q*^+^/*T* range, the activation energy of structural relaxation decreases from ~320 to 175 kJ·mol^−1^.

With the knowledge of the activation energy value, the curve-fitting approach (based on the algorithm reported in [57]) can be used to estimate the other three TNM parameters. As the pre-exponential factor A_TNM_ is only related to the position of the relaxation peak on the temperature axis, its determination is straightforward; for the average value obtained in the low-*q*^+^ region of CR cycles, i.e., Δh* = 275 kJ·mol^−1^, the corresponding *A_TNM_* = 10^−42.6^. Contrary to the pre-exponential factor, the *β*&*x* values are responsible for the actual shape of the relaxation peak, and their determination via the direct curve-fitting approach is thus sensitive to even the slightest distortions of the DSC signal. In the case of TA Instruments DSCs, such distortion is commonly observed and is likely associated with the thermal inertia phenomenon [58]. For this reason, an alternative and robust simulation-comparative method [59] was employed to determine *β* and *x* from the CBZ relaxation data. The simulation-comparative method does not rely on the entire course of the DSC signal throughout the entire glass transition range (as in curve-fitting) but instead evaluates only the more robust maximum height of the normalized relaxation overshoot, *C_p_^max^*. In particular, the *C_p_^max^* quantity is evaluated for a series of CHR cycles heating scans, and the data are plotted as a function of log(*q*^−^/*q*^+^). Similar dependences are then evaluated for CHR data series simulated for different combinations of TNM parameters *β* and *x* (with known ∆h* and *A_TNM_*); the best match then corresponds to the sought *β* and *x* values. In the present case, the *β*-*x* hyperspace was mapped with a resolution of 0.01 in the 0.2–1.0 range for both parameters. The experimental heating scans data obtained within the CHR cycles (and normalized according to Equation (2)) are shown in the middle graph in Figure 5. The heights of the relaxation peaks determined from this graph are then shown in the bottom Figure 5 graph, together with the simulated *C_p_^max^*-log(*q*^−^/*q*^+^) dependences. Note that only every tenth simulated dependence is depicted for better clarity. The best correlation between the experimental and simulated *C_p_^max^*-log(*q*^−^/*q*^+^) dependences (determined via the least squares method; the corresponding sum of squared residue was 0.00923) was obtained for *β* = 0.57 and *x* = 0.44.

A comparison of the CBR relaxation parameters with the TNM results obtained for other drugs, similarly classified according to Taylor [14], is shown in Table 2.

In absolute values, the TNM parameters obtained for CBZ are in line with results reported for other similarly classified APIs. Notably, a relatively strong linear correlation is observed between Δh* and *β*, with a correlation coefficient of *r* = −0.984. Compared to the other listed drugs, CBZ exhibits the lowest degree of structural heterogeneity (associated with the highest *β* value), indicating a typologically and density-wise more uniform span of relaxation motions, as expressed through the narrower distribution of relaxation times. This is in good correspondence with a higher *x* value, which can be inversely related [42] to the degree of cooperativity during the structural relaxation. The structural uniformity and lower degree of cooperativity within the relaxing CBZ glassy matrix are thus also reflected in the relatively low activation energy value.

### 3.2. Cold Crystallization Kinetics

**Figure 6:**
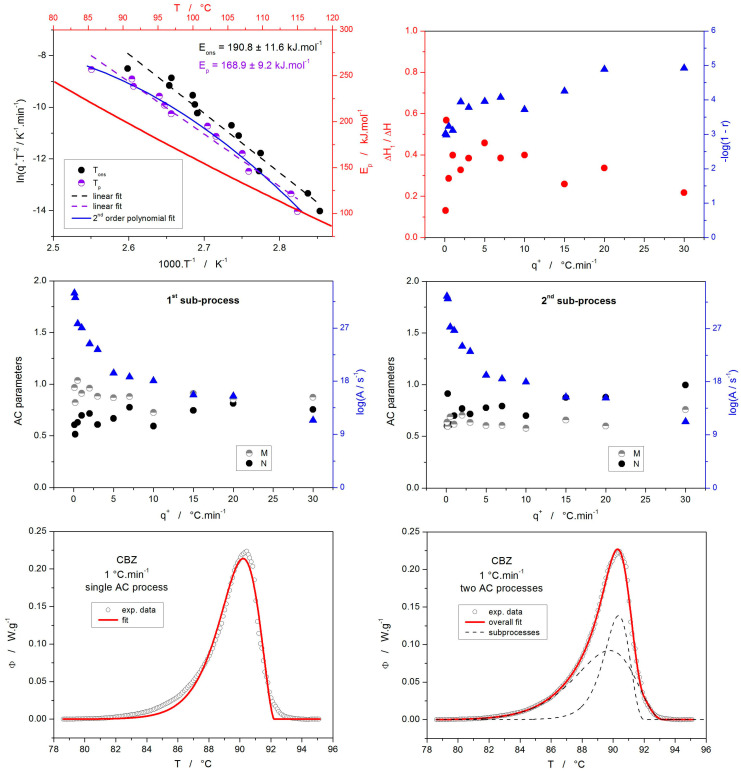
(**Top left graph**): Kissinger evaluation of *E* using the temperatures corresponding to the crystallization peak onset and maximum with the listed values corresponding to the linear fits (bottom and left axes); *E*-*T* dependence determined from the *T_p_* values fit by the 2nd-order polynomial (top and right axes). (**Top right and middle graphs**): *q*^+^ dependences of kinetic parameters determined by means of the sc-MKA method. (**Bottom graphs**): Example crystallization signal fit by either one or two AC processes.

The description of the CBZ crystallization data (shown in Figure 1) was conducted in accordance with the standard approach for the glassy materials, i.e., within the framework of the solid-state equation that separates the temperature dependence of crystal growth (rate constant dependent only on temperature) and its autocatalytic features (kinetic model with the degree of conversion α being the only variable) [46]:

(9) $$\Phi =∆H\times \frac{d\alpha }{dt}=∆H\times K\left(T\right)\times f\left(\alpha \right)=∆H\times A\times e^{-E/RT}\times f(\alpha )$$

(10) $${f(\alpha )}_{AC}=\alpha^{M}{\left(1-\alpha \right)}^{N}$$

where *Φ* is the DSC heat flow signal, Δ*H_c_* is the crystallization enthalpy, *A* is the pre-exponential constant, *E* is the activation energy of crystallization, *R* is the universal gas constant, and *f*(*α*) is a function defining the shape of the crystallization peak. For the crystallization processes initiated from glassy matrices, one of the most flexible and most often used models is the autocatalytic (AC) Šesták–Berggren model [60] (Equation (10)) with two kinetic exponents *M* and *N*. Similarly to the kinetic analysis of structural relaxation, also the kinetic analysis of the crystallization processes (enumeration of Equations (9) and (10)) starts with the determination of the energy activation E. Traditionally, E is evaluated by means of the Kissinger method [61]:

(11) $$ln\left(\frac{q^{+}}{T_{p}^{2}}\right)=-\frac{E}{RT_{p}}+const.$$

where *T_p_* is the temperature corresponding to the maximum of the crystallization peak. Technically, the same evaluation can be applied to any other characteristic temperature that is evaluated with sufficient precision and represents a state with constant conversion α. For the present data, the Kissinger method was applied to the *T_p_* as well as *T_ons_* data from Table 1; the results are shown in the top left graph of Figure 6. Since both dependencies are evidently curved, exhibiting temperature-dependent kinetics, the *E* values obtained via linear fitting should be viewed only as a rough estimate. A much more accurate evaluation can be obtained by interpolating the dependence using a second-order polynomial function—its derivation can then be used to calculate the *E*-*T* dependence (which is, for the present CBZ data shown in correspondence to the top and right axes of the top left graph in Figure 6). Note that very similar values (lower by ~10 kJ·mol^−1^) of *E* were also obtained by means of the Augis–Bennett method [62].

With knowledge of the *E*-*T* dependence, the relatively recently developed method of single-curve multivariate kinetic analysis (sc-MKA) [63] can be used to determine the remaining parameters from Equations (9) and (10). The standard MKA method [64] is based on the following set of equations:

(12) $$RSS=\sum_{j=1}^{n}\sum_{{First}_{j}}^{{Last}_{j}}w_{j,k}{\left({Yexp}_{j,k}-{Ycal}_{j,k}\right)}^{2}$$

(13) $$w_{j}=\frac{1}{{\left|{\left[d\alpha /dt\right]}_{max}\right|}_{j}+{\left|{\left[d\alpha /dt\right]}_{min}\right|}_{j}}$$

where *RSS* is the sum of squared residue, *n* is a number of measurements, *j* is an index of the given measurement, *First_j_* is the index of the first point of the given curve, *Last_j_* is the index of the last point of the given curve, *Yexp_j,k_* is the experimental value of the point *k* of curve *j*, *Ycal_j,k_* is the calculated value of the point *k* of curve *j* (calculated in accordance with Equation (9)), and *w_j_* is a weighting factor for curve *j*. In the sc-MKA method, *E* is fixed at the independently determined value (e.g., by the Kissinger method), which negates the need to evaluate multiple curves simultaneously. Each curve is thus processed separately, providing a unique set of kinetic parameters, which can then be plotted in dependence on *T* or *q*^+^. From the mathematical point of view, the first summation in Equation (12), as well as the necessity for employment of the weighting factor *w_j_*, are eliminated. The non-linear optimization is based on the Levenberg–Marquardt algorithm, with the objective function being the minimized sum of squared residues.

Apart from the base process kinetics represented by Equations (9) and (10), a concrete reaction mechanism often needs to be considered in the case of complex phase transformations. Since the fits of the CBZ crystallization data were rather unsatisfactory using a single AC process (see the bottom graphs in Figure 6), an overlap of two independent crystallization processes had to be used to properly reflect the shape of the crystallization peak; this changes the related mathematics to:

(14) $$\frac{d\alpha_{x}}{dt}=A_{x}\times exp\left(-\frac{E_{x}}{RT}\right)\times \alpha_{x}^{M}\times {\left(1-\alpha_{x}\right)}^{N}$$

(15) $$1=\sum_{x=1}^{n}\alpha_{x}$$

where “*x*” is the index of the given individual sub-process, and “*n*” is the number of sub-processes (two in the present case). Note that the independent sub-process nature is common and plausible for the multistep crystallization that originates from a frozen-in glassy matrix with individually formed nuclei. The employment of two sub-processes led to a practically perfect description of the experimental data—see the bottom row in Figure 6 for a visual example; the full set of correlation coefficients (expressed as “−log(1 − *r*)” is also shown in Figure 6). Since the crystallization peak preserves its uniform nature and does not split into two (nor does it exhibit any recognizable shoulders), an identical *E* value can be considered for both crystallization sub-processes. Hence, the *E*-*T* dependence determined via the Kissinger method was used within the sc-MKA framework to determine the *q*^+^ trends in the individual kinetic parameters—see Figure 6. The main purpose of these dependencies lies in the possibility of extrapolating the trends to the “zero” heating rate, i.e., isothermal conditions. This approach was proven to provide significantly more accurate kinetic predictions [63,65].

The most relevant kinetic predictions apply to amorphous drugs, specifically those that consider the crystallization rate near the *T_g_*. Since the glass transition is the breakpoint for several crystal growth mechanisms to either cease or accelerate, the temperature is used in practice to consider different processing routes for the amorphous phase. For CBZ, the upper *T_g_* limit appears to be ~60 °C (see Figure 2). By extrapolating the kinetic trends depicted in Figure 6 to the lowest *q*^+^ and using the *E* value obtained from the data-curve measured at 0.1 °C·min^−1^, a 300 min isotherm at 60 °C predicts only 0.05% crystallinity, i.e., CBZ is very stable at *T* ≤ *T_g_*, and processing at *T_g_* (such as, e.g., self-healing of the quench-induced surface defects) is practically risk-free.

### 3.3. Thermal Decomposition Kinetics

**Figure 7:**
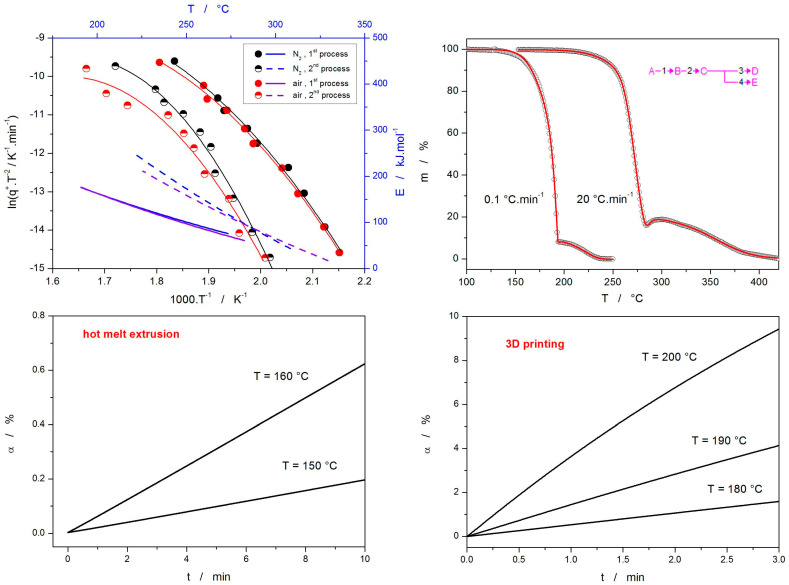
(**Top left graph**): Kissinger evaluation of E using the temperatures corresponding to the TGA points of inflection determined from the derivative TGA data for the two step-like mass losses (bottom and left axes); *E*-*T* dependences determined from the Kissinger dependences fit by the 2nd-order polynomials (top and right axes). (**Top right graph**): Example TGA data (measured under N_2_ atmosphere) fit by the sc-MKA method employing the depicted reaction scheme. (**Bottom graphs**): Kinetic predictions calculated using the kinetic parameters determined for the 0.1 °C·min^−1^ TGA data-curve obtained under N_2_ atmosphere—the predictions were calculated for isothermal annealing at denoted temperatures characteristic for the processes of pharmaceutical hot melt extrusion and 3D printing.

The thermal decomposition of CBZ was processed kinetically, similar to cold crystallization. The base set of solid-state equations, i.e., Equations (9)–(13), was employed. The Kissinger analysis is illustrated in the top-left graph of Figure 7. Apart from significant curvatures indicating the need for second-order polynomial fits, the most important information is that the activation energy of the dominant (low-*T*) decomposition step is practically identical for both atmospheres at low *q*^+^. This means that the kinetic predictions extrapolated to lower *T* (than the measured experimental value) will also be identical, considering the general similarity of the decomposition processes under both atmospheres (see Figure 3). The Kissinger plots in Figure 7 are combined with the graph showing the evaluated *E*-*T* dependencies, confirming the identical *E* values for the first, or dominant, decomposition step. Note that this step is denoted as “first”, whereas the smaller, consequent series of sub-processes is denoted as a “second” process. As was already mentioned in Section 2, the decomposition exhibits a complex behavior, where the first (dominant) decomposition step is followed by the second step, which, however, shows a temporary mass increase before the sample is completely gasified (sample mass is reduced to zero). The iterative kinetic analysis has shown that this behavior needs to be modeled as a four-step reaction mechanism—see the top right graph of Figure 7.

The first, dominant decomposition step is modeled by two consecutive AC reactions; the second, smaller decomposition then has to be modeled as two competitive reactions (a mass loss and a mass gain). The following set of kinetic and balance equations is thus used:

(16) $$\frac{dm_{A}}{dt}={-A}_{1}\times exp\left(-\frac{E_{1}}{RT}\right)\times m_{B}^{M_{1}}\times {\left(1-m_{B}\right)}^{N_{1}}$$

(17) $$\frac{dm_{B}}{dt}=A_{1}\times exp\left(-\frac{E_{1}}{RT}\right)\times m_{B}^{M_{1}}\times {\left(1-m_{B}\right)}^{N_{1}}-A_{2}\times exp\left(-\frac{E_{2}}{RT}\right)\times m_{C}^{M_{2}}\times {\left(1-m_{C}\right)}^{N_{2}}$$

(18) $$\frac{dm_{C}}{dt}=A_{2}\times exp\left(-\frac{E_{2}}{RT}\right)\times m_{C}^{M_{2}}\times {\left(1-m_{C}\right)}^{N_{2}}-A_{3}\times exp\left(-\frac{E_{3}}{RT}\right)\times m_{D}^{M_{3}}\times {\left(1-m_{D}\right)}^{N_{3}}\;-A_{4}\times exp\left(-\frac{E_{4}}{RT}\right)\times m_{E}^{M_{4}}\times {\left(1-m_{E}\right)}^{N_{4}}$$

(19) $$\frac{dm_{D}}{dt}=A_{3}\times exp\left(-\frac{E_{3}}{RT}\right)\times m_{D}^{M_{3}}\times {\left(1-m_{D}\right)}^{N_{3}}$$

(20) $$m_{E}=1-m_{A}-m_{B}-m_{C}-m_{D}$$

(21) $$m=m\left(t=0\right)+∆m\times m_{rel}\left(t\right)$$

(22) $$m_{rel}=w_{fol1}\times \left(1-m_{A}\right)+w_{fol2}\times \left(1-m_{A}-m_{B}\right)+\left(1-w_{fol1}-w_{fol2}\right)\times \;\left(w_{com3}\times m_{D}+w_{com4}\times \left(1-m_{A}-m_{B}-m_{C}-m_{D}\right)\right)$$

where *m_x_* is the normalized mass of the given phase (as identified according to the scheme shown in Figure 7); the normalization is carried out with respect to the overall sample mass. Hence, *m_x_* is effectively equal to the partial degree of conversion *α_x_*.

Examples of the resulting sc-MKA fits (with the proper *E*-*T* dependences employed) are given in the top right graph of Figure 7; the full set of the kinetic parameters obtained via the sc-MKA analyses is shown in Table 3. Due to the monotonous trends observed for the dominant process (which is crucial during the low-*T* extrapolation of the kinetic behavior), kinetic predictions can be made based on the set of kinetic results obtained from the TGA curve measured at 0.1 °C·min^−1^. Such predictions, calculated for isothermal annealing at different temperatures, are shown in the bottom two graphs of Figure 7. The lower set of temperatures is relevant for the pharmaceutical hot melt extrusion [66], where the commonly used polymeric matrix—the hydroxypropylmethylcellulose (HPMC) under the brand name Affinisol [67,68]—requires ~150–160 °C for the filaments to be printed with consistent diameter and for the API to be homogeneously dispersed within the filament. Exposure to these temperatures during hot melt extrusion usually lasts a few minutes, which is, however, still safe, as only a few tenths of a percent of the sample will decompose during that time. On the contrary, a different situation arises for pharmaceutical 3D printing, where filaments made from Affinisol require temperatures of ~190 °C. The printing times (exposure to the high temperature) are usually cumulatively < 30 s, but even such short times can lead to 1–2% of the sample being decomposed. This would be particularly problematic if the decomposition led to toxic products. The bottom-right graph is also relevant with respect to the preparation of the amorphous phase using the melt–quench method. In the present study, 20 s at 200 °C were used in the melt phase, which (realistically) translates into 15 s of the true exposition to this temperature, as the absorbed heat is during the first seconds consumed to increase the sample temperature (after the first exposition to 200 °C), and to melt the sample at *T_m_*. Nonetheless, even such a short exposition could have still led to ~1% of CBZ being degraded. Note that during the melt–quench optimization, the *T*/*t* conditions were chosen so that the sample remained visually unchanged, i.e., the color of the melt was still clear, without any yellow tint. Most importantly, the low CBZ thermal stability appears to represent a significant problem for its potential mass production by means of the melt–quench process.

### 3.4. Correlation Between the Relaxation and Crystallization Kinetics

**Figure 8:**
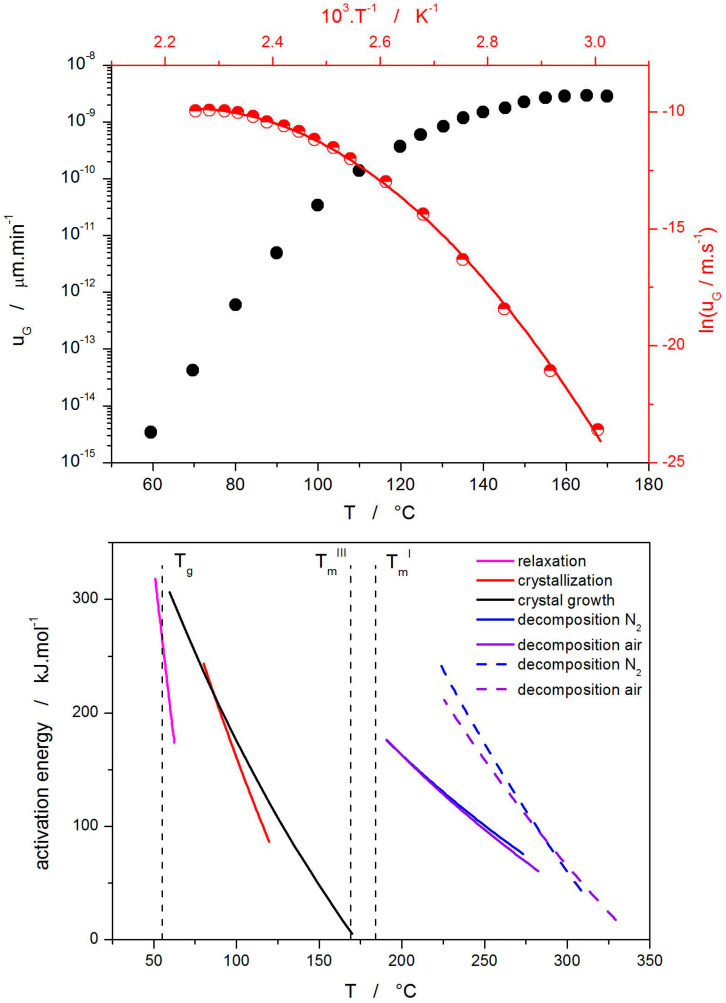
(**Top graph**): Crystal growth rate data taken from [25] and transformed to evaluate the activation energy *E_G_*. (**Bottom graph**): Activation energies determined for various thermally induced processes occurring in (amorphous) CBZ. “Crystallization” and “crystal growth” denote the data from DSC and microscopy, respectively.

To evaluate the mutual relationship between structural relaxation and crystal growth, both perspectives on the latter process need to be considered. The microscopically observed CBZ crystal growth rate data were taken from [25]—see Figure 8 for their depiction. The activation energy of the microscopic manifestation of crystal growth, *E_G_*_,_ can be calculated according to [69]:

(23) $$\frac{dln\left(u_{G}\right)}{d\left(1/T\right)}=-\frac{E_{G}}{R}$$

where *u_G_* is the crystal growth rate. The E_G_ evaluation is shown in the top graph of Figure 8, scaled according to the top and right axes. The comparison of all relevant activation energies determined within the framework of the present study is shown in the bottom graph in Figure 8. The key temperature region is below the melting temperatures of the two CBZ forms. As is apparent, the activation energy for crystal growth increases with decreasing *T*, where at *T_m_*, *E_G_* ≈ 0, while at *T_g_*, *E_G_* > 300 kJ·mol^−1^. The course of the *E_G_*-*T* dependence is also confirmed through the present macroscopic monitoring of crystal growth in melt-quenched bulk samples. Interestingly, and contrary to the other amorphous drugs [40,41,42,43] recently studied in this regard (indomethacin, nimesulide, nifedipine, griseofulvin), only CBZ exhibits at *T_g_* significantly higher activation energy for crystal growth/crystallization E compared to the activation energy of structural relaxation Δh*. The *E* = Δh* occurs slightly below *T_g_*. Out of the five above-mentioned amorphous APIs, CBZ is the only one that does not exhibit a pronounced tendency toward the sub-*T_g_* crystal growth modes (surface-enhanced growth and glass–crystal growth). At the same time, CBZ is the only drug (out of these five), borderline classified in class 2 according to Taylor [14], exhibiting a higher crystallization tendency than the others. Hereby, we propose that the significantly lower Δh* enables relaxation movements on the laboratory timescale within a wider temperature range, which in turn disrupts the sub-*T_g_* crystal growth modes typical of low-molecular organic glasses. In addition, the increased mobility of the amorphous phase may also be responsible for the higher crystallization tendency of CBZ, as well as for its ability to primarily form the crystalline phase within the bulk volume of the (undercooled) melt. This proposition appears to be novel and the first of its kind; hence, it is only speculative. Nonetheless, since the relationship between crystal growth modes and structural relaxation movements is a popular yet unresolved topic, we believe it is worth pursuing further.

## 4. Materials and Methods

**Scheme 1:**
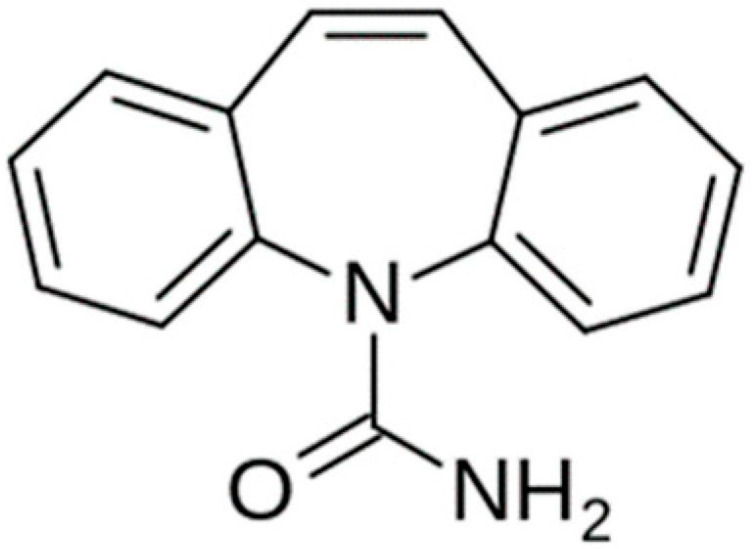
Molecular structure of carbamazepine.

The amorphous carbamazepine (CBZ; the corresponding molecular structure is shown in Scheme 1) was prepared using a single-sample melt–quench technique. Approximately 2 mg (accurately weighed to 0.01 mg) of crystalline CBZ (purity > 99%; Sigma-Aldrich, Prague, Czech Republic) was introduced directly into the DSC pan, which was then placed on a temperature-regulated hotplate. The CBZ melted for 20 s at 200 °C. Consequently, the DSC pan containing the molten CBZ sample was quenched via a brass heat sink annealed at 0 °C. In this way, a fully amorphous sample was always obtained. However, despite the placement of the crystalline sample on one of the sides of the DSC pan, the rapid melting led to the irreproducible formation of a few droplets in the middle of the DSC pan bottom (in addition to the main bulk of the molten sample being positioned at the bottom pan edges owing to its surface tension). Each sample was prepared just before the corresponding DSC measurement and immediately placed into the DSC cell. In this way, the maximum reproducibility of the amorphous phase preparation was ensured.

The DSC measurements were performed using a heat flow differential scanning calorimeter (DSC Q2000, TA Instruments, New Castle, DE, USA), equipped with an autosampler, an RCS90 cooling accessory, and T-zero technology. The DSC calibration was conducted on the basis of the melting temperatures and enthalpies of In, Zn, and H_2_O standards measured at a heating rate *q*^+^ = 10 °C∙min^−1^. Note that the time delay of the DSC response was measured to show that it is negligible compared to the present measurement and reproducibility errors—the onset temperatures of the In melting peak were: 156.16 °C (*q*^+^ = 0.1 °C∙min^−1^), 156.24 °C (*q*^+^ = 1 °C∙min^−1^) and 156.52 °C (*q*^+^ = 10 °C∙min^−1^), i.e., ~0.35 °C over the two orders of magnitude of the q^+^ span. This would translate into the increases in Δh* from CR cycles (Figure 5) by ~10 kJ·mol^−1^, and *E* values for the crystallization (Figure 6) and decomposition (Figure 7) by ~5 kJ·mol^−1^. As the shift in the In *T_m_ons_* occurs primarily at higher *q*^+^, it does not affect any reported kinetic predictions, which were carried out based on the activation energies calculated for the low-*q*^+^ data. The DSC samples were measured in hermetically sealed low-mass Al pans, i.e., in a static air atmosphere. For the macroscopic crystallization, a series of simple heating scans were performed in the 0–210 °C temperature range at the following heating rates: *q*^+^ = 0.1, 0.2, 0.5, 1, 2, 3, 5, 7, 10, 15, and 20 °C∙min^−1^ (always with freshly prepared sample). The structural relaxation measurements were performed using standard constant ratio (CR) [56] and constant heating rate (CHR) [47] cyclic methods, where the sample is periodically cooled and heated through the glass transition range. During the CR cycles, the ratio between the cooling and consequent heating rate is constant (usually *q*^−^/*q*^+^ = 1); during the CHR cycles, the heating rate is constant (usually *q*^+^ = 10 °C·min^−1^), and only the cooling rates vary. For the present measurements, the following series of cooling rates were applied: *q*^+^ = 0.5, 1, 2, 3, 5, and 10 °C∙min^−1^.

The decomposition of CBZ was performed using the STA (joint DTA+TGA) 449 F5 Jupiter instrument (Netzsch, Selb, Germany) equipped with a DSC/TG holder. The measurements were performed on the original as-purchased crystalline CBZ powder in either an air or N_2_ atmosphere; the purge gas flow was 50 mL·min^−1^. The sample masses were ~2–3 mg (accurately weighted to 0.01 mg), and the measurements were performed in open Al pans. Similarly to the crystallization measurements, the CBZ thermal decomposition was also measured by means of simple heating scans in the 30–400 °C temperature range at *q*^+^ = 0.1, 0.2, 0.5, 1, 2, 3, 5, 7, 10, and 20 °C∙min^−1^.

The bonding arrangements in the glassy as well as crystalline CBZ samples were explored by means of Raman microscopy, using the Raman microscope DXR2 (Nicolet, Thermo Fisher Scientific, Prague, Czech Republic), equipped with a 785 nm excitation diode laser (30 mW, laser spot size of 1.6 μm) and CCD detector. The experimental setup for the Raman experiments consisted of a 20 mW laser power on the sample, a 1 s duration for a single scan, and 20 scans summed into a single spectrum. The morphology of the formed crystallites was observed using an optical microscope (iScope PLMi, Euromex, Arnhem, The Netherlands; transmission mode), equipped with 40× and 80× high-quality objectives and a Moticam visual camera.

## 5. Conclusions

Complete thermokinetic characterization of amorphous carbamazepine was performed by means of thermo-analytical, spectroscopic, and microscopic techniques; this research was dominantly focused on the kinetics of the processes associated with the amorphous phase (structural relaxation and cold crystallization) and CBZ thermal stability (decomposition kinetics).

Regarding the amorphous state, CBZ is classified as a borderline amorphizable API, which was confirmed already during the melt–quench preparation, where it was possible to prepare fully amorphous only relatively low sample amounts (~2 mg)—this led to the necessity of preparing a sample for each thermokinetic measurement separately (very good repeatability of the melt–quench was required and achieved). The structural relaxation process exhibited standard degrees of non-linearity and non-exponentiality (TNM parameters: *β* = 0.57, *x* = 0.44), indicating the mediocre levels of uniformity and cooperativity of the relaxation motions. However, the activation energy of structural relaxation was found to be quite low, decreasing from ~310 kJ·mol^−1^ at 50 °C to ~175 kJ·mol^−1^ at 62 °C. These values are unusually significantly lower compared to the activation energies of both microscopically and macroscopically recorded crystal growth within the glassy matrix, which for CBZ was >320 kJ·mol^−1^ at *T_g_*. A proposition regarding the conversely related activation energies of relaxation and crystal growth being responsible for the absence of sub-*T_g_* crystal growth modes was made. The crystallization kinetics, despite the DSC signal manifesting as a single exothermic peak, were described using two fully overlapping autocatalytic processes with temperature-dependent activation energies as well as the degree of autocatalysis. Based on this description, the kinetic predictions were calculated for the crystallization rate at *T_g_*—CBZ, which was found to be very stable, allowing for processing of the amorphous phase (e.g., self-healing of the quench-induced mechanical defects or internal stress) at these temperatures.

On the other hand, the thermogravimetric measurements of CBZ have shown that it is quite unstable even below *T_m_*, which greatly limits the possibility of mass-producing amorphous CBZ via melt-quenching. The thermal decomposition proceeded almost identically in the N_2_ and air atmospheres. A complex reaction mechanism, consisting of a series of consecutive and competing reactions, was proposed to explain the second decomposition step, which exhibited a temporary mass increase. Whereas a negligible degree of CBZ degradation was predicted for the temperature characteristic for the pharmaceutical hot-melt extrusion (~150 °C), the risk of CBZ degradation during the pharmaceutical 3D printing is considerably higher (1–2% mass loss at temperatures 190–200 °C).

## Figures and Tables

**Table 1:** Base thermal characteristics evaluated from DSC curves depicted in Figure 1. *T_ons_* and *T_p_* refer to the extrapolated onset and maximum of the crystallization peak; *T_m_ons_* and *T_m_p_* denote the same for the melting peak.

*q*^+^/°C.min^−1^	*T_g_*/°C	*T_ons_*/°C	*T_p_*/°C	Δ*H_c_*/J.g^−1^	*T_m_ons_*/°C	*T_m_p_*/°C	Δ*H_m_*/J.g^−1^
0.1	50.67	77.32	81.03	47.48	183.45	184.68	89.96
0.2	48.61	79.28	82.08	45	187.17	188.57	87.85
0.5	51.58	87.51	89.28	45.21	188.13	189.23	97.7
1	49.53	87.24	90.42	53.42	187.96	189.37	113.3
2	53.54	91.06	95.07	56.3	188.14	189.68	110.6
3	54.27	92.31	96.59	57.05	187.96	189.96	108.6
5	56.92	98.45	103.3	60.57	188.31	190.57	100.1
7	57.33	98.93	104.59	63.32	188.1	190.58	113.3
10	59.36	99.3	105.55	61.1	188.16	190.48	107.4
15	58.87	103.67	110.52	61.72	187.92	191	108.4
20	59.42	103.27	110.83	64.72	187.96	191.4	114.4
30	61.16	111.67	118.92	66.93	187.91	192.16	102.3

**Table 2:** TNM relaxation parameters obtained for various amorphous drugs.

Drug	∆h*/kJ·mol^−1^	*β*/-	*x*/-	Reference
Carbamazepine	275	0.57	0.44	This work
Indomethacin	308	0.53	0.32	[34]
Nimesulide	305	0.54	0.40	[43]
Griseofulvin	379	0.41	0.29	[41]
nifedipine	279	0.55	0.38	[40]

**Table 3:** Sc-MKA parameters obtained for the thermal decomposition of CBZ in N_2_ atmosphere.

*q*^+^/°C·min^−1^	0.1	0.2	0.5	1	2	3	5	7	10	20
log(*A*_1_/s)	16.68803	15.55479	13.99215	12.78519	10.64098	10.07923	8.59189	8.25742	7.74375	5.75816
*E*_1_	174.74	166	153.9	144.55	126.1	120.4	106.56	102.8	94.4	77.15
*N*_1_	1.51103	0.74778	0.77778	0.26916	0.15355	3.85 × 10^−2^	1.02 × 10^−2^	5.01 × 10^−3^	0.53381	0.93172
*M*_1_	2.52 × 10^−2^	2.32 × 10^−4^	1.01 × 10^−4^	6.21 × 10^−5^	5.89 × 10^−5^	5.31 × 10^−5^	0.10877	0.15823	0.33233	0.32967
log(*A_2_*/s)	16.12644	15.33341	14.11392	13.13384	11.00083	10.43413	8.85628	8.56524	7.72344	6.32128
*E*_2_	174.74	166	153.9	144.55	126.1	120.4	106.56	102.8	94.4	77.15
*N*_2_	0.20443	0.32288	0.48118	0.55288	0.54284	0.54883	0.4046	0.36066	0.31932	1.41584
*M*_2_	0.46254	0.59597	0.64207	0.63111	0.61939	0.59092	0.55085	0.59061	0.65583	0.57754
log(*A*_3_/s)	24.40883	21.32904	18.44007	15.97648	15.453	13.99532	11.60053	9.17964	8.62322	3.92277
*E*_3_	245.1	222	197.4	173.8	168.1	154.3	132.31	107.3	96.28	43.95
*N*_3_	3.15329	1.77813	2.38565	2.24905	2.24681	1.9831	2.39261	1.98374	2.83091	5.36013
*M*_3_	0.63821	0.61661	0.61352	0.69363	0.61634	0.64226	0.64775	0.59279	0.86141	0.67531
log(*A*_4_/s)	22.42912	19.74473	16.78756	14.30939	13.79004	12.41094	10.18802	7.8666	6.29772	1.96211
*E*_4_	245.1	222	197.4	173.8	168.1	154.3	132.31	107.3	96.28	43.95
*N*_4_	1.2522	0.82817	1.20213	1.11947	1.11085	1.0086	1.02698	1.1933	1.00453	0.95017
*M*_4_	0.18135	0.13964	0.14512	0.14421	0.12459	9.14 × 10^−2^	6.68 × 10^−2^	2.37 × 10^−2^	0.17295	0.54406
*w_fol1_*	0.28799	0.27388	0.20953	0.2526	0.24408	0.18507	0.17136	0.18327	0.18492	0.15229
*w_fol2_*	0.63209	0.61135	0.65112	0.60612	0.58186	0.56257	0.61504	0.46183	0.66541	0.71104
*w_com3_*	−0.65184	−0.26457	−0.42554	−0.34494	−0.43478	−0.7266	−1.39134	−1.40146	−0.38187	−3.00739
*w_com_*_4_	8.5935	9.73956	10.01935	9.58223	10.81768	10.80582	7.92621	8.90405	12.47581	3.24355
*m*/%	−103.904	−103.52	−104.332	−103.243	−103.755	−106.6	−97.5805	−108.022	−104.85	−104.349

## Data Availability

The data are available in a FigShare repository at https://doi.org/10.6084/m9.figshare.29205917; accessed on 25 June 2025.

## Supplementary Materials

The following supporting information can be downloaded at: https://www.mdpi.com/article/10.3390/ijms26136136/s1: DSC calibration measurements (Figures S1–S3), comparison of identical CR and CHR cycles (Figure S4).
