# Supplementary material for: Kinetics of Phase Transitions in Amorphous Carbamazepine: From Sub-Tg Structural Relaxation to High-Temperature Decomposition
Source: Int J Mol Sci. 2025 Jun 26;26(13):6136. doi: 10.3390/ijms26136136 (PMC12250088; doi:10.3390/ijms26136136)
Supplement: Supplementary file 1 [file ijms-26-06136-s001.zip › ijms-3705296-supplementary.pdf]

Supplemental online material

1) The supplementary data below show the measurements of the indium melting peak (with the extrapolated onset evaluated) measured at different heating rates.

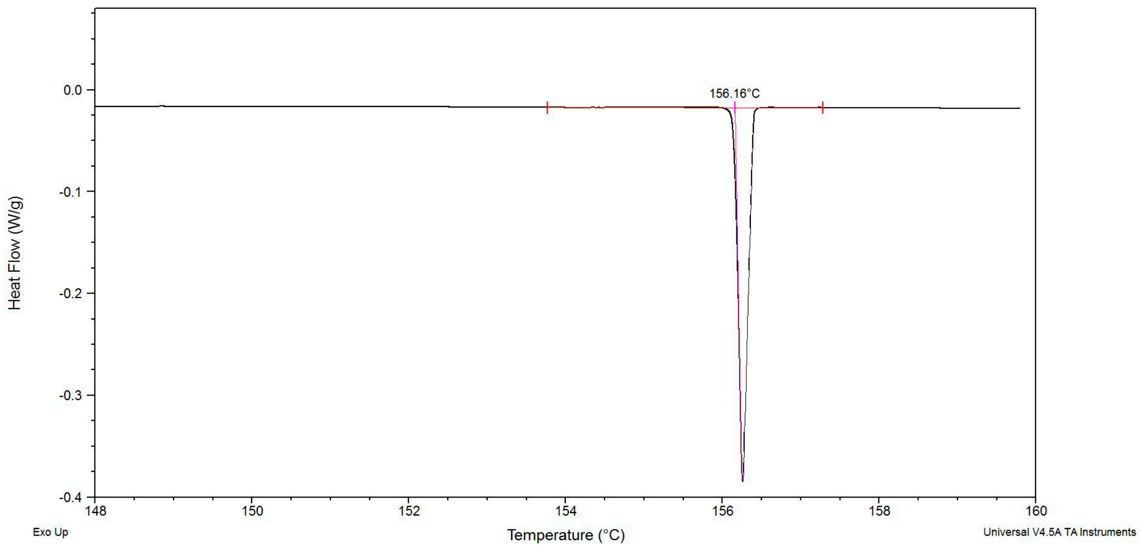

Fig. S1: DSC measurement performed at 0.1 °C·min^{-1}.

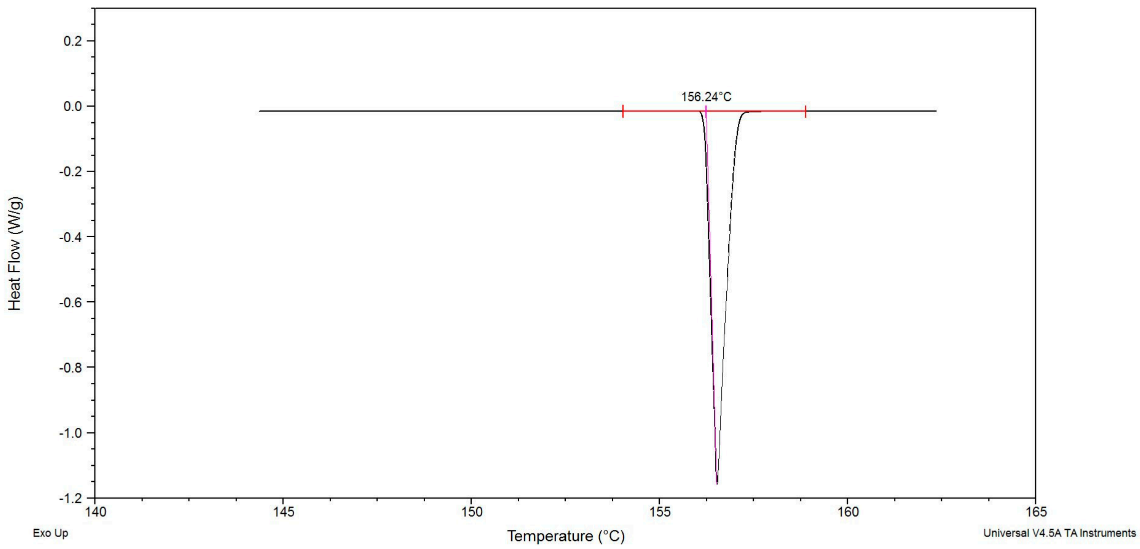

Fig. S2: DSC measurement performed at 1 °C·min^{-1}.

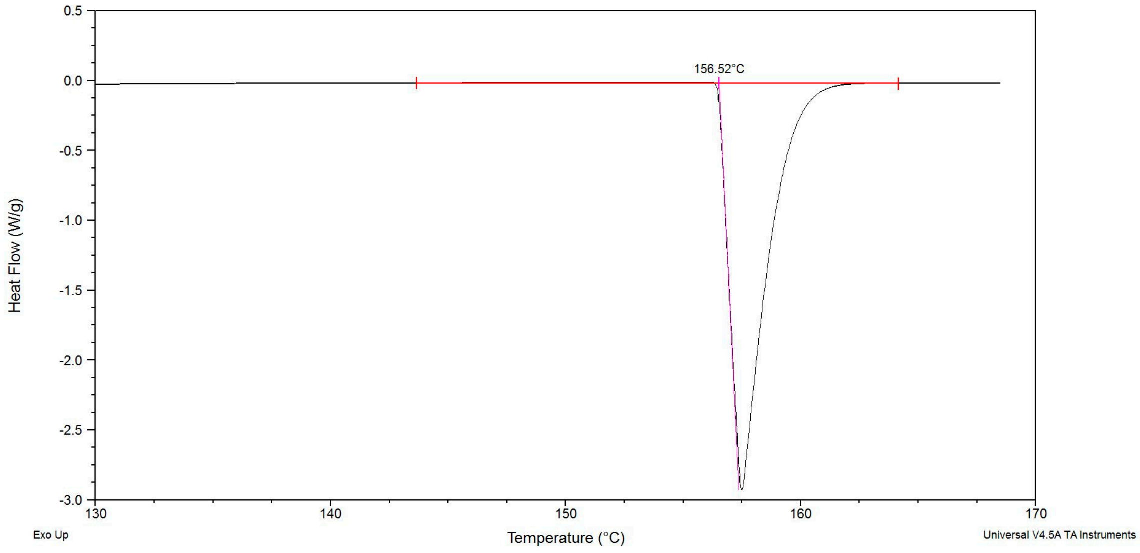

Fig. S3: DSC measurement performed at 10 °C·min^{-1}.

2) The supplementary data below show the comparison of the identical CR and CHR heating scans.

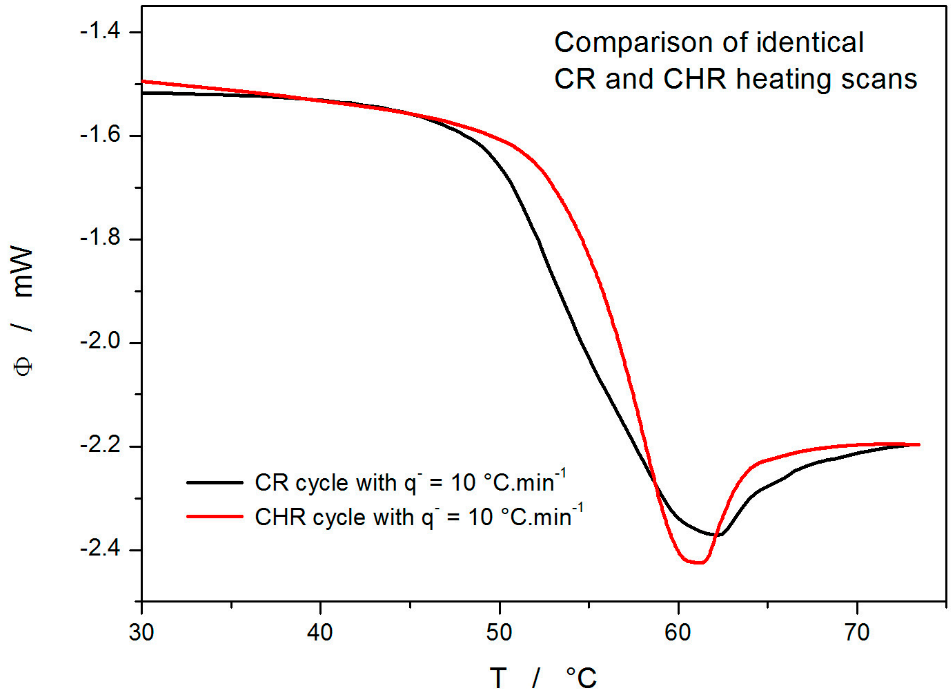

Fig. S4: Comparison of the identical CR and CHR heating scans performed within the "q^- = 10 °C·min^{-1} & q^+ = 10 °C·min^{-1}" cycle.
